# The Enhancement of Leak Detection Performance for Water Pipelines through the Renovation of Training Data

**DOI:** 10.3390/s20092542

**Published:** 2020-04-29

**Authors:** Tu T.N. Luong, Jong-Myon Kim

**Affiliations:** 1 Department of Computer Engineering, University of Ulsan, Ulsan 44610, Korea; ngtu.mta@gmail.com; 2 School of IT Convergence, University of Ulsan, Ulsan 44610, Korea

**Keywords:** intelligent leak detection, acoustic emission signals, statistical parameters, support vector machine, wavelet denoising, Shannon entropy

## Abstract

Leakage detection is a fundamental problem in water management. Its importance is expressed not only in avoiding resource wastage, but also in protecting the environment and the safety of water resources. Therefore, early leak detection is increasingly urged. This paper used an intelligent leak detection method based on a model using statistical parameters extracted from acoustic emission (AE) signals. Since leak signals depend on many operation conditions, the training data in real-life situations usually has a small size. To solve the problem of a small sample size, a data improving method based on enhancing the generalization ability of the data was proposed. To evaluate the effectiveness of the proposed method, this study used the datasets obtained from two artificial leak cases which were generated by pinholes with diameters of 0.3 mm and 0.2 mm. Experimental results show that the employment of the additional data improving block in the leak detection scheme enhances the quality of leak detection in both terms of accuracy and stability.

## 1. Introduction

Leakage detection is a primary problem in water management [1,2]. About 20–30% of the water has been lost in water supply system every year. Especially, the loss of water can be up to 50% in some systems [2]. The growing demand for water inspires reconsideration of the management and supply of pipeline systems. Complications in exploiting new water bodies can be beaten by decreasing water losses [3]. Furthermore, the present attention of environmental protection and issues related to water quality encourages a growing interest in leakage detection. The community concerned with water resources has been concentrated more on the natural environment. However, the guardianship of water against incursions in pipes and the protection of the environment from the arrival of a transported contaminant are as significant as the protection of aquifers and well-fields from contaminant discharge [4]. Subsequently, methodologies for early leak detection are strongly urged. Additionally, they should not induce the interruption of piping actions, and they should be simple enough to actualize in practice.

Many studies on leak detection for water supply systems have been conducted and published. The avenues may be passive or active [5], and hardware-based or software-based [6]. Passive methods require direct visual investigation or supervision of sites, while active methods include a signal analysis. Signals used in active methods can be acoustic, vibration, flow rate, or pressure. Besides, hardware-based methods are classified depending on the type of special sensing devices such as acoustic monitoring, vibration analysis, cable sensor, etc. On the other hand, software-based methods are categorized based on the type of software programs and techniques used for leak detection such as support vector machine, harmonic wavelet analysis, genetic algorithm, etc. Among those avenues, acoustic emission (AE)-based methods, which are passive and hardware-based, are auspicious, since AE sensors can quickly recognize small leaks, offering high sensitivity in relation to fault buildup in a piping system. Accordingly, AE-based methods for pipeline diagnostics have been exploited [7,8,9,10,11,12].

In recent years, defect diagnosis methods based on modelling have been extensively used to improve the availability and reliability of mechanical systems subject to defects [13,14,15]. These avenues use high-dimensional signature vectors to prevent the hazards of dropping likely essential information. Nevertheless, some defect signatures are repetitious or inapplicable to the predicting models (namely unsupervised and supervised learning). As a result, these defect signatures can be a fundamental source of diagnostic efficiency deterioration. To address this issue, discriminative defect signature selection has turned into an indispensable part of trustworthy diagnosis. Basically, the subsequent two steps are carried out in the signature selection procedure, namely a configuration step of signature subsets and an assessment step of signature subset quality. Specifically, a number of signature subsets are first assembled and then assessed. Based on the assessment step, signature selection strategies are fundamentally assorted into wrappers or filters. Filter avenues use an assessment strategy that is separated from any classification strategy, while wrapper strategies employ accuracy estimates for particular classifiers during the evaluation of signature subset quality [16]. As a result, wrapper methodologies give better diagnostic efficiency for predetermined classifiers than filter strategies, theoretically. Nevertheless, filter avenues are computationally profitable because they bypass the accuracy estimation step for a specific classifier.

Practically, various conditions influence leakage signals, such as pipe diameter, surrounding environment, pipeline material, flow rate, and pressure [17,18]. Therefore, the data collected for training classifiers may not be large enough and extracted features from it may not be smooth enough to cover the whole probability space of features. As a result, the accuracy of feature evaluation and selection based on these data may be reduced. Furthermore, leak detection is a real application, and thus the techniques should be simple, effective, and easy to implement by hardware. Recently, Tu et al. offered an effective multivariable signature assessment coefficient (MSAC) to simultaneously evaluate the interclass separability and intraclass compactness depending on predicting the signature space from a restricted data point number [12]. Based on this coefficient, the diagnostic performance, in case the training data is not broad enough, is considerably improved. Nevertheless, the quality of leak detection is also affected by outsiders. These effects are regarded as noise data points with a low probability distribution, and they are far from the central data point in the same class. The accuracy and stability of a model greatly depends on the training dataset. If the training dataset is less generalized, the diagnostic model built on it will have reduced reliability and stability of performance. To deal with this problem, a data renovation method was introduced in this study. Particularly, the MSAC was first used to evaluate signatures as a filter method, and then the most discriminative signature subset was produced. Based on the selected signature subset, detecting and removing outsiders from the known dataset before training a diagnostic model is a key issue.

Once the discriminatory feature subset is determined and the known dataset is renovated, they are further employed to train a Support Vector Machine (SVM) classifier, which is a supervised model with higher accuracy than unsupervised models such as *k*-NN classifier, and with faster processing speed and lower hardware requirements than deep learning. In this work, the offered method was used to detect artificial leaks created in a laboratory with hole diameters of 0.3 mm and 2.0 mm.

The organization of this paper is as follows. The offered method is presented in Section 2. The data collecting method for leak detection is illustrated in Section 3. The efficacy of the proposed method is validated in Section 4, and the final section shows the conclusions.

## 2. The Offered Method

**Figure 1:**
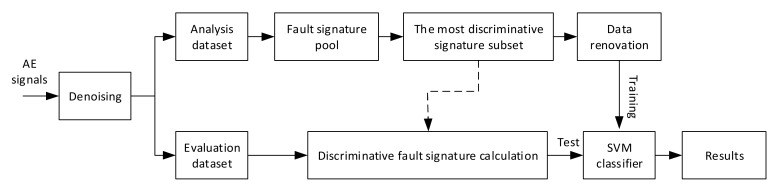
The overall flow diagram of the leak detection model.

The overall flow diagram of leak detection is illustrated in Figure 1. First, the acquired AE signals were denoised by a Wavelet algorithm based on normalized Shannon entropy, which was also adopted in some recent studies of leak detection [12,19,20]. After that, the denoised signals were divided into separate analysis and evaluation datasets. The isolation of the evaluation dataset from the analysis dataset was to ensure the reliability of the performance evaluation results. Based on the analysis dataset, a defect signature pool was configured and the most discriminative signature subset, which was also applied on the evaluation dataset, was determined. Subsequently, based on selected features, the analysis dataset was renovated by detecting and removing outsiders before it was used to train SVM classifiers. Finally, the efficacy verification of the proposed method was carried out on the evaluation dataset. Each specific part is described in detail as follows.

### 2.1. Noise Reduction Using a Wavelet Transform and Shannon Entropy

Due to the nature of the AE mechanism, leakage noise is commonly nonstationary [21,22]. Time-frequency analysis methods, which are powerful tools to analyze the time-varying nonstationary signals, are recommended to study a signal in both the time and frequency domain simultaneously. Many studies have adopted the wavelet transform to detect the leak by reason of its multiresolution capability [23,24,25].

A form of wavelet transform which allows multiresolution investigation is known as a Wavelet packet transform (WPT) [26]. Signals can be decomposed into both wavelet coefficients and the scaling values through the WPT technique. Based on this technique, the complete decomposition hierarchy is provided. As a result, because of uniform frequency secondary groups, the decomposition becomes extremely adoptable [27].

A signal $\psi \left(t\right)$ with a fixed energy, which is expressed as a mother wavelet, is a consecutive vacillating function of intensely short duration as indicated in Equation (1):

(1) $$\psi_{s,\tau }(t)=\frac{1}{\sqrt{s}}\psi (\frac{t-\tau }{s}),s>0;-\infty <\tau <\infty ,$$

where $\psi_{s,\tau }\left(t\right)$ consists of the total standardized expressions (expansions) in time *t* designated by s>0 (scale factor) and translation in time *t* is designated by −∞<τ<∞. Equation (2) expresses a cross correlation of *x(t)* with $\psi_{s,\tau }\left(t\right)$ which depicts the Wavelet transformation of a signal *x(t)* [24,27,28,29]. Mathematically, the similarity between two signals can be identified by cross-correlation analysis. Given two sets of signals $x_{i}$ and $y_{i}$, where i=0,1,2,…,N−1, Equation (3) describes the function of normalized cross correlation with zero time-lag. The normalized cross correlation is a numerical quantity between 0 and 1, which predicts the closeness in characterization between two signals. Two signals which have identical characterizations generate a normalized cross correlation coefficient of 1.0 [30]:

(2) $$WT_{x}(s,\tau )≜\int x(t)\psi_{s,\tau }(t)dt,$$

(3) $$R=\frac{\sum x_{i}y_{i}}{(\sum x_{i}^{2}{)}^{\frac{1}{2}}(\sum y_{i}^{2}{)}^{\frac{1}{2}}},$$

The determination coefficient is made by executing the WPT with filter banks through recursive schemes. Low-frequency components (approximations) and high-frequency components (details) at each resolution level are obtained by transmitting the signal *x(t)* to a two-channel filter. Compared to the wavelet transform technique, which decomposes only the approximations, the WPT technique decomposes both details and approximations at every resolution level.

**Figure 2:**
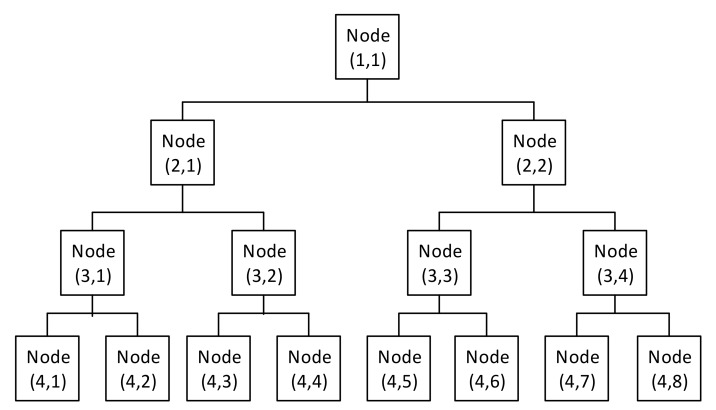
The binary tree of the organization and numbering of sub-bands in discrete wavelet packet transform (DWPT).

The most indispensable challenge in wavelet analysis is the selection of the mother wavelet function as well as the decomposition level of signal. Among orthogonal wavelets, Daubechies (DB) wavelets have been widely implemented, as they match the transient components in acoustic and vibration signals [31]. The order of the mother wavelet function and the level of decomposition were often determined by trial-and-error methods based on intrinsic characteristics of the data [31,32]. In this study, the selected mother function is DB15, and the number of levels was experimentally determined by Equation (3). Figure 2 illustrates the binary hierarchical tree of discrete wavelet packet transform (DWPT) coefficients. Each node of this tree was considered as a sub-band and numbered according to its level and its ordinal in level. Here, hierarchical levels and ordinals are numbered from 1.

An algorithm based on informative entropy was utilized to detect the unnecessary signatures in an AE signal acquired during a test, where the informative entropy was considered a cost function. In this method, only the sub-bands which focus the major information carried by the signal are intended to be picked. Generally, the following equations denote the Shannon entropy $H\left(X_{j}\right)$ if $X_{j}=\left(x_{j},k\right)$ is a cluster of coefficients of a specified sub-band of the WPT tree at stage of resolution *j*:

(4) $$H(X_{j})=-\sum_{k}P_{k}\ln(P_{k}),$$

(5) $$P_{k}=\frac{{\left|x_{j,k}\right|}^{2}}{{\left\|X_{j}\right\|}^{2}},$$

Here, ${\left\|X_{j}\right\|}^{2}=\sum_{k}x_{j,k}^{2}$ signifies a norm of $X_{j}$ [26]. A large value of $H\left(X_{j}\right)$ means that the signal is in higher disorder and carries less information. As a result, the corresponding sub-band and its subordinates are discarded. This implies that the entropy computes a correlation of energy among the sub-bands. At this moment, the aim is to select the WPT branch which transports the minor disorders and has minimum conceivable energy. If the informative entropy of the current resolved sub-band is smaller than that of the subsequent resolved sub-band, then the total data is conserved. Otherwise, a lesser energy level of resolution is essential. In other words, the selected sub-band should have the lowest entropy value and the highest resolution level. After that, the preferred sub-bands are used to reconstruct the AE signal such that the most significant part of the signal is saved, and the complementary component which is known to be noise is removed.

### 2.2. Fault Signature Pool Configuration

According to the authors of [11,12], intelligent leak detection schemes are well corroborated with statistical parameters from the time and frequency domains. Thus, this study used them as fault signatures for the identification of leaks. Statistical parameters for the given one-second AE data, *x(n)*, are defined in Table 1 and Table 2. These parameters were computed in the frequency and time domain, and involved the peak ($sp_{1}$), the root-mean-square ($sp_{2}$), kurtosis ($sp_{3}$), crest-factor ($sp_{4}$), impulse factor ($sp_{5}$), shape factor 1 ($sp_{6}$), skewness ($sp_{7}$), the square-mean-root ($sp_{8}$), margin factor ($sp_{9}$), peak-to-peak ($sp_{10}$), kurtosis factor ($sp_{11}$), energy ($sp_{12}$), clearance factor ($sp_{13}$), shape factor 2 ($sp_{14}$), the fifth normalized moment ($sp_{15}$), the sixth normalized moment ($sp_{16}$), entropy ($sp_{17}$), spectral centroid ($sp_{18}$), the root-mean-square of frequency ($sp_{19}$), root variance of frequency ($sp_{20}$), and the frequency spectrum energy ($sp_{21}$).

**Figure 3:**
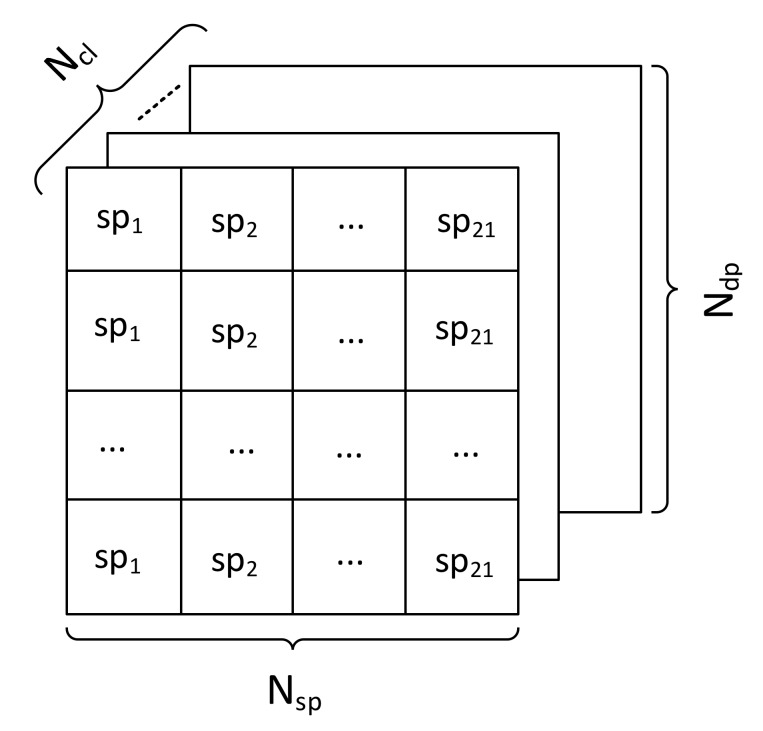
The illustration of data point configuration.

In summary, the dimensionality of the fault-signature pool used in the feature selection process is $N_{dp}\times N_{sp}\times N_{cl}$, where $N_{dp}$, $N_{sp}$, $N_{cl}$ are the number of data points per leak condition class in the analysis dataset, the number of statistical parameters, and the number of classes to be discriminated in this study, respectively. Figure 3 illustrates an example of a data point configuration used to yield the most discriminatory feature subset. The set of elements in the fault-signature pool is denoted by $X=\left\{x\left(dp,sp,cl\right)\right\}$, with *dp* = 1, …, $N_{dp}$, *sp* = 1, …, $N_{sp}$, and *cl* = 1, …, $N_{cl}$. Variables dp, sp, cl represent coordinates of data point *x* in the dataset *X*.

### 2.3. The Generation of the Discriminative Signature Subset

In order to achieve fairness, statistical parameters need to be standardized before evaluating and grading. This study used a simple scaling method with the following formula:

(6) $$\tilde{x_{i}}=\frac{x_{i}-min\left(X_{k}\right)}{max\left(X_{k}\right)-min\left(X_{k}\right)},$$

Here, $X_{k}=\left\{\left.x_{i}\right|sp=k\right\}$, ${\tilde{X}}_{k}=\left\{\left.{\tilde{x}}_{i}\right|sp=k\right\}$ denote original and standardized sets of values of the $k^{th}$ signature (i.e., $k^{th}$ statistical parameter) respectively. After standardization, values of different signatures were all in the range [0,1].

To solve the small dataset problem, Tu et al. recently introduced an MSAC to evaluate the discrimination of fault signatures between two different classes [12]. The MSAC method estimates the potential value range of $k^{th}$ dimension of the signature sub-space of class i by interval $\left[mean\left(X_{k}^{i}\right)-3std\left(X_{k}^{i}\right),\;mean\left(X_{k}^{i}\right)+3std\left(X_{k}^{i}\right)\right]$, where $X_{k}^{i}$ denotes the set of values of $k^{th}$ signature of data points in class i. Therefore, the crossing level between two signature sub-spaces of classes i,j at dimension k which is denoted by $MSAC_{k}^{i,j}$ is determined by Equation (7):

(7) $$MSAC_{k}^{i,j}=1-\frac{3\left(std\left(X_{k}^{i}\right)+std\left(X_{k}^{j}\right)\right)}{\left|mean\left(X_{k}^{i}\right)-mean\left(X_{k}^{j}\right)\right|},\;X_{k}^{i}=\left\{x|sp=k,cl=i\right\},X_{k}^{j}=\left\{x|sp=k,cl=j\right\},$$

Values of $std\left(X_{k}^{i}\right)$, $std\left(X_{k}^{j}\right)$ represent the intraclass compactness of classes, and $\left|mean\left(X_{k}^{i}\right)-mean\left(X_{k}^{j}\right)\right|$ represents the interclass separability. According to the authors of [12], the bigger the MSAC, the better discrimination. Thus, MSAC expresses the distinguishable ability of signatures for each pair of classes. Although it is simple and has low computing cost, it is still effective and suitable for real applications such as leak detection. In this paper, the MSAC was used to rank signatures from top to bottom and the discriminatory signature subset was created by picking the signatures on top.

### 2.4. Data Renovation

To build a classification model, the correctness and generalization of the training dataset are extremely important. If the dataset is inaccurate or not generalized, then the accuracy, reliability, and stability of the trained model may be reduced. Related studies have most focused on big data [33,34,35]. Meanwhile, the problem with leak detection using a smart fault diagnostic model is related to the small data problem, because leakage signals are affected by many external factors. Therefore, it is necessary to revamp the dataset. In machine learning, the quality of samples is more important than their quantity, especially when the quantity is not large. The higher quality the samples, the greater the generalization ability and the better the accuracy. In a class, points that are far from the center and have a low probability distribution are known as outsiders. They are less significant than the rest and may be noise points. Consequently, they should be detected and removed.

**Figure 4:**
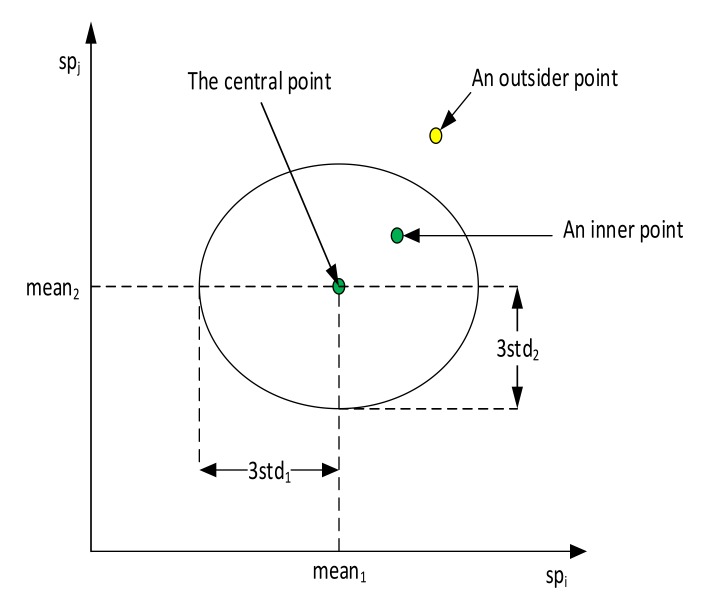
The illustration of how to classify data points.

This study focuses on improving the quality of data before training the classification model with a simple and effective technique. This technique includes three processes of detecting, eliminating outsiders, and updating dataset alternately until there no longer exist outsiders in the renovated dataset. In this study, we assumed that the statistical parameter values were Gaussian random variables. In term of statistics, the probability that each statistical parameter value in a specific class lies in the interval $\left[mean-3std,mean+3std\right]$ is equal to 99.73% [36], where *mean* is the mean and *std* is the standard deviation of their values in that class. This study used such range as the limit for outsider detection to ensure that outsiders were both far from central points and had a low probability distribution. Outsiders were defined as data points that were outside the confident interval (CI), which was determined through the central coordinate (CC) (i.e., the central point) and the standard deviation of each dimension (i.e., each statistical parameter or signature) of the signature space. Figure 4 illustrates an example about how to identify the central point, inner points, and outsider points in a signature space having two dimensions.

**Figure 5:**
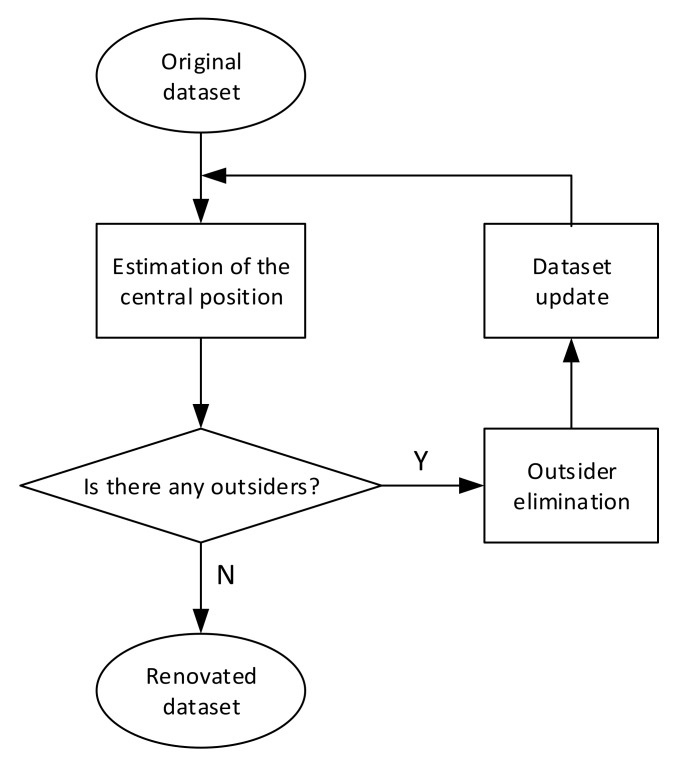
The process of improving the training dataset.

Denote $X_{k}^{i}=\left\{x|sp=k,cl=i\right\}$ as the set of values of $k^{th}$ statistical parameter of all data points in class *i*. The CC of class *i*, $CC_{i}$, is defined in Equation (8). The CI of value of $k^{th}$ statistical parameter (i.e., $k^{th}$ dimension) of data points in the signature space of class i is given in Equation (9). A data point is considered as an outsider if any dimension of that data point is outside the CI of such dimension. The process of improving the dataset was implemented separately for each class and illustrated in Figure 5. Whenever outsiders are detected and eliminated, the dataset needs to be updated. After that, values of CC and CIs also needs to be updated, and as a result, new outsiders can be detected and eliminated. This process ends when no outsider is detected in the updated dataset:

(8) $${\mathrm{CC}}_{i}=\left(\mathrm{mean}\left(X_{1}^{i}\right),\;\mathrm{mean}\left(X_{2}^{i}\right),\ldots ,\;\mathrm{mean}\left(X_{N_{sp}}^{i}\right)\right),$$

(9) $$CI_{k}^{i}=\left[mean\left(X_{k}^{i}\right)-3std\left(X_{k}^{i}\right),\;mean\left(X_{k}^{i}\right)+3std\left(X_{k}^{i}\right)\right],$$

### 2.5. Classification

This study used a two-class SVM classifier whose theory is based on the idea of structural hazard minimization [37]. In the SVM method, the generalization error is minimized and the geometric margin between two classes is maximized. This method is also known as the maximum margin classifier. In this study, the kernel function was used to map the input data into a high-dimensional signature space and detect the best hyper plane to discriminate between the two classes of input data. The margin between two classes in the feature space was maximized by the best hyper plane. This quadratic optimization problem was worked out using Lagrange multipliers. The term “support vectors” is used to refer to the points which are nearest to the optimal hyper plane for each class [38]. Support vectors are selected along the surface of a kernel function which can be chosen among different functions such as polynomial, linear, radial-based, and sigmoid for the SVM during the training phase [39]. Based on a set of predetermined support vectors that are members of the set of training inputs, SVM distributes data with two class labels.

Kernel function parameter selection is one of the significant details of SVM modeling. In this paper, we used the radial based function (RBF), which is a common kernel function that can be employed to any sample distribution through parameter selection. The RBF has been used more and more in the nonlinear mapping of SVMs. The RBF kernel function expression is:

(10) $$K(x_{i},x_{j})=\exp\left(-\gamma {\left\|x_{i}-x_{j}\right\|}^{2}\right),$$

The corresponding minimization problem of an SVM is expressed below:

(11) $$\underset{\alpha_{i}}{\min}\frac{1}{2}\sum_{i=1}^{n}\sum_{j=1}^{n}y_{i}y_{j}\alpha_{i}\alpha_{j}\exp\left(-\gamma {\left\|x_{i}-x_{j}\right\|}^{2}\right)-\sum_{i=1}^{n}\alpha_{i},\sum_{i=1}^{n}y_{i}\alpha_{i}=0,\;0\leq \alpha_{i}\leq C,$$

The minimum value of Equation (11) depends on the choice of parameters $\left(C,\gamma \right)$. In this study, the grid search method was used to get the final optimal parameters $\left(C,\gamma \right)$ [40]. This method respectively takes *m* values in *C* and takes *n* values in γ, for the m×n combinations of $\left(C,\gamma \right)$, trains different SVM respectively, then estimates the learning precision. We can obtain the highest study accuracy of the best combination as the optimal parameters in the m×n combinations of $\left(C,\gamma \right)$.

## 3. Experiment Setup

**Figure 6:**
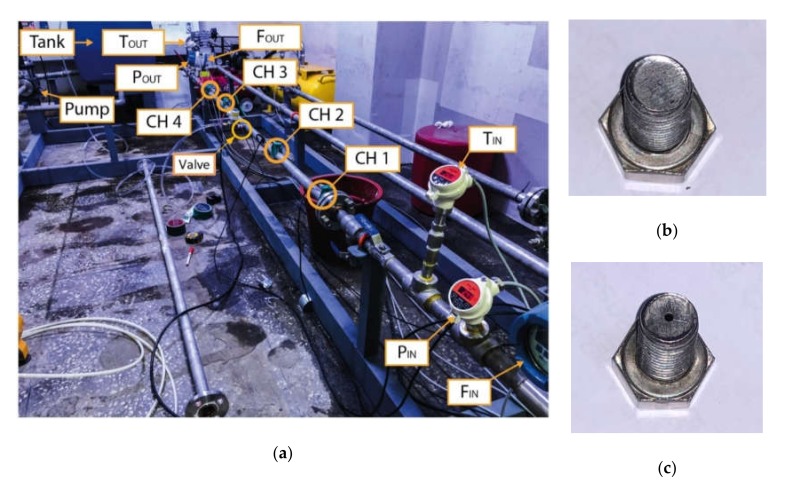
AE signal acquisition from a water pipeline system: (**a**) The test pipeline and component arrangement; (**b**) the pinhole size of 0.3 mm; (**c**) the pinhole size of 2.0 mm.

Figure 6 shows the setup of the AE signal acquisition from a water pipeline system. The pipe, which was made of stainless steel 304, had an outside diameter of 34 mm and a wall thickness of 3.38 mm. A pump was employed to keep the water flow constant at a pressure of 3 bar. The experiments were executed under a balanced temperature of approximately 29 °C. AE sensors were mounted on both sides of the testing pipe fragment. The distance from sensors to the leak position was 1000 mm. In this study, wideband differential-auto sensor test (WDI-AST) sensors were used to provide high sensitivity and a wide frequency band. The characteristics of the sensors are recapped in Table 3.

The experiments were based on two leak cases with different pinhole diameters, i.e., 0.3 mm and 2.0 mm, which were considered dataset 1 and 2, respectively. The AE signals were collected in one-second time lengths and sampled at a frequency of 1 MHz. Details of the datasets which were used to assess the offered method are described in Table 4. In this table, “normal” means the no leakage case. Since different datasets were acquired in different dates, and operating conditions such as temperature, pressure, flow rate, etc. have impacts on AE signals, each “normal” data is taken accordingly with the leak data to have coherence with background condition.

## 4. Results and Discussion

**Figure 7:**
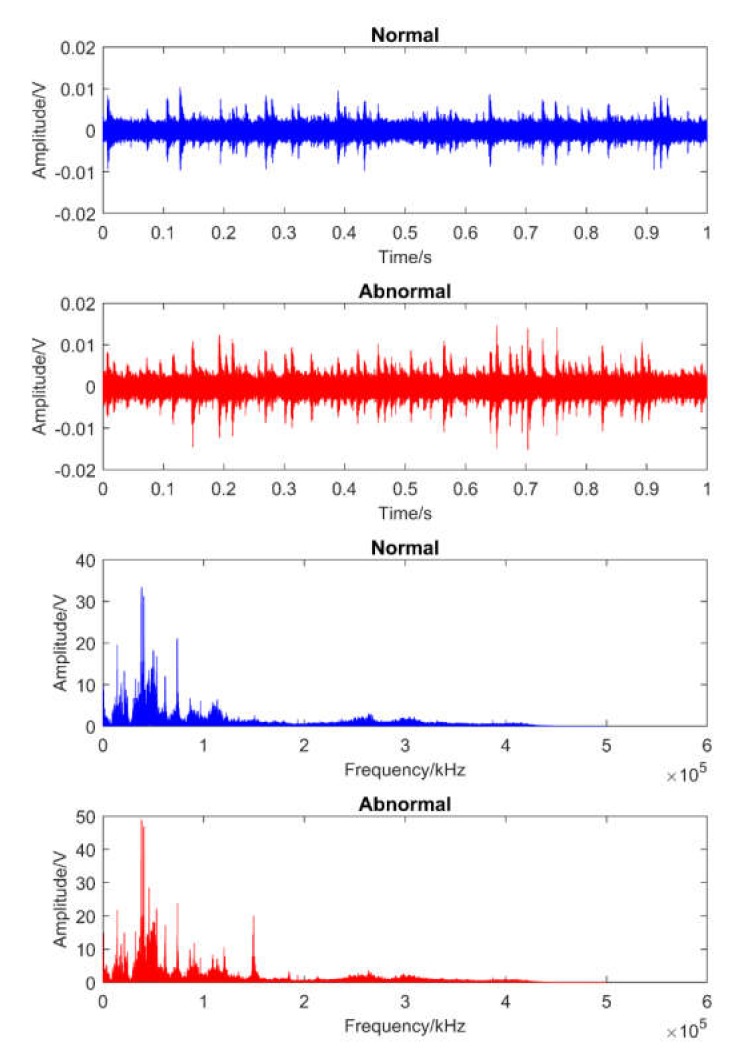
The illustration of the obtained AE signals in dataset 1 over the time and frequency domains.

**Figure 8:**
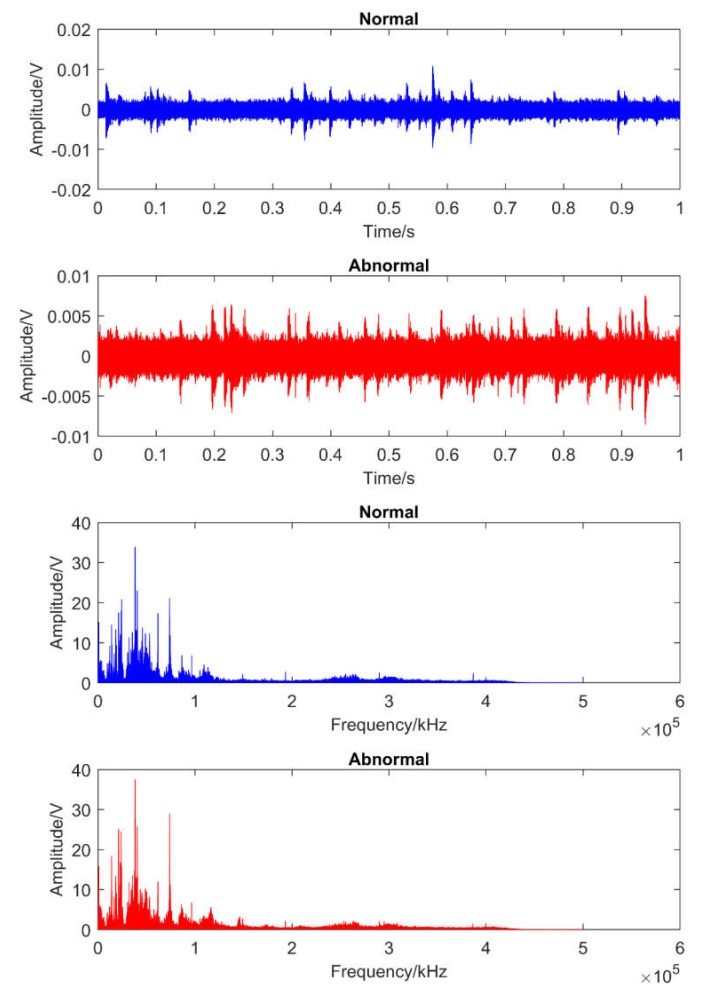
The illustration of the obtained AE signals in dataset 2 over the time and frequency domains.

Figure 7 and Figure 8 illustrate one obtained AE signal sample of each case for each dataset over the time and its fast Fourier transform in frequency domains. It is clear that these original signals contained noise, and that there was not much difference between signals at healthy and unhealthy states.

**Figure 9:**
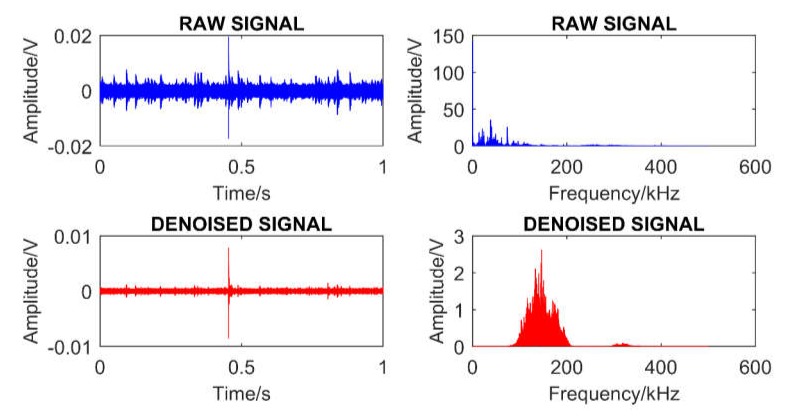
Differences between the signals before and after filtering noise in both the time and frequency domains.

To extract the most informative part of the signals, sub-bands were first produced by implementing the DWPT on each raw AE signal. Then, the optimal sub-band was selected depending on the minimum wavelet entropy before being employed to restore the AE signal. Figure 9 shows the difference between signals before and after denoising in both the time and frequency domains.

In the next step, the fault signature pool was created from reconstructed AE signals in the analysis dataset. Then, the MSAC was used to evaluate the signatures. Table 5 lists signatures in order of best to worst in terms of leak detection, together with their MSACs corresponding to each case.

**Figure 10:**
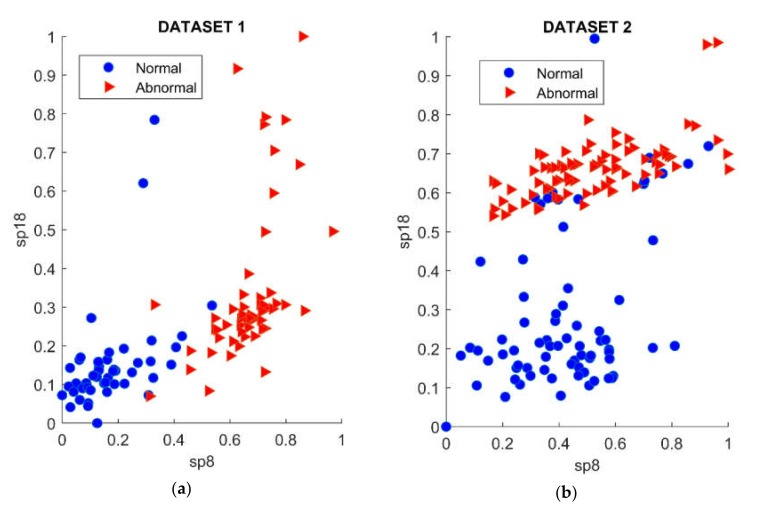
The distribution of data points according to the selected features corresponding to each leak case: (**a**) Leak size of 0.3 mm; (**b**) leak size of 2.0 mm.

After that, the two best signatures on top were selected as a discriminatory feature subset. In such a manner, the feature sub-set that was most discriminative for both cases included one parameter on the time domain, namely the square-mean-root, and one parameter on the frequency domain, namely the spectral centroid. Figure 10 illustrates the distribution of data points according to the selected features corresponding to each leak case. It can be seen that data points in the same class, in the case of a lower-level leak (pinhole size of 0.3 mm), had a higher concentration than in the case of a higher-level leak (pinhole size of 2.0 mm), while the separation between classes in the first case was lower than the other. The reason for this may be that instability of the AE signal increased belong with the leakage level. It follows that the leak detection method of using statistical parameters of AE signals was limited by leak level in both directions. Specifically, the greater the leakage level, the lower the concentration level in the same class and the greater the interclass separability.

To enhance the stability and quality of the SVM classifiers, the training dataset needs to be improved by detecting and removing outsiders, which may be noise data points because of their low probability distribution and weak generalization. Based on the renovated analysis dataset, the SVM classifiers were trained before being used to detect leaks in the evaluation dataset. To evaluate the proposed method, this study used a 10-fold cross validation to compare classification accuracies (CAs). The CA given in Equation (12) is the ratio between the number of correctly classified data points (i.e., true points), $N_{TP}$, and the total of data points, $N_{total}$. The results of CAs of three methods are shown in Table 6. Here, “All” represents the conventional method, which uses all of 21 fault signatures without signature selection and data renovation, whereas the conventional method [12] uses a signature selection with MSAC without data renovation. In general, the proposed method, which added the data enhancement block, outperformed the method in [12], which had the same signature subset. Specifically, the former had no worse results than the latter in 10 total assessments of both cases. In addition, the former surpassed the latter by four times in dataset 1 and three times in dataset 2. It follows that the former enhanced the average CA of 4.61% and 1.58% compared to the latter when datasets 1 and 2 were used, respectively. Therefore, it is proven that the proposed method is both more accurate and more stable than the previous method.

(12) $$CA=\frac{N_{TP}}{N_{total}}\times 100\%$$

Compared to the method of using all the signatures in terms of CA, the proposed method was better in dataset 1, but worse in dataset 2. However, the proposed method, which used only two features, significantly reduced the number of dimensions of the fault signature vector compared to the non-signature-selection method, which used 21 features. This means that it is possible to mitigate the computational responsibility in the configuration of signature vectors in real applications. Moreover, low-dimensional signature vectors can assist in reduction of consumed time to train classifiers. Table 7 shows computational time comparison between the proposed and conventional method which employed all 21 signatures. Compared to the conventional method, the processing speed of the proposed method was improved by 31.17% in training, 76.77% in test, and 40.14% in total for the dataset 1. Similarly, those improvements for the dataset 2 were 41.63%, 76.80%, and 48.63% respectively. All experiments were implemented with MATLAB R2018b on an Intel Core i7-7700 CPU operating at 3.60 GHz.

## 5. Conclusions

In this paper, an intelligent leak detection method based on a model using statistical parameters extracted from AE signals was used for early leak detection. Since leak signals depend on many operation conditions, the training data in real-life situations usually has a small size. To solve the problem of a small dataset, a data improving method based on enhancing the generalization ability of the data was proposed. To evaluate the effectiveness of the proposed method, this study used the datasets obtained from two artificial leak cases which were generated by pinholes with diameters of 0.3 mm and 0.2 mm. Experimental results showed that the employment of the additional data improving block in the leak detection scheme enhances the quality of leak detection in both terms of accuracy and stability.

## Figures and Tables

**Table 1:** General statistical parameters in time-space of acoustic emission (AE) signals.

$sp_{1}=\max\{\left|x\right|\}$	$sp_{2}=\sqrt{\frac{1}{N}\sum_{i=1}^{N}x_{i}{}^{2}}$	$sp_{3}=\frac{1}{N}{\sum_{i=1}^{N}\left(\frac{x_{i}-mean(x)}{std(x)}\right)}^{4}$
$sp_{4}=\frac{\max\{\left|x\right|\}}{rms(x)}$	$sp_{5}=\frac{peak(x)}{\frac{1}{N}\sum_{i=1}^{N}\left|x_{i}\right|}$	$sp_{6}=\frac{rms(x)}{\frac{1}{N}\sum_{i=1}^{N}\left|x_{i}\right|}$
$sp_{7}=\frac{1}{N}{\sum_{i=1}^{N}\left(\frac{x_{i}-mean(x)}{std(x)}\right)}^{3}$	$sp_{8}={\left(\frac{1}{N}\sum_{i=1}^{N}\sqrt{\left|x_{i}\right|}\right)}^{2}$	$sp_{9}=\frac{peak(x)}{smr(x)}$
$sp_{10}=\max\{\left|x\right|\}-\min\{\left|x\right|\}$	$sp_{11}=\frac{1}{rms{\left(x\right)}^{3}}$	$sp_{12}=\sum_{i=1}^{N}x_{i}{}^{2}$
$sp_{13}=\frac{peak(x)}{smr(x)}$	$sp_{14}=\frac{pp(x)}{\frac{1}{N}\sum_{i=1}^{N}\left|x_{i}\right|}$	$sp_{15}=\frac{1}{N}{\sum_{i=1}^{N}\left(\frac{x_{i}-mean(x)}{std(x)}\right)}^{5}$
$sp_{16}=\frac{1}{N}{\sum_{i=1}^{N}\left(\frac{x_{i}-mean(x)}{std(x)}\right)}^{6}$	$sp_{17}=-\sum_{i=1}^{N}p_{i}\log_{2}p_{i}$	

$x=\left[x_{1},\;x_{2},\ldots ,x_{N}\right]$ denotes a signal in time-space, $mean\left(x\right)=\frac{1}{N}\sum_{i=1}^{N}x_{i}$, $s\mathrm{td}\left(x\right)=\sqrt{\frac{1}{N}\sum_{i=1}^{N}{\left(x_{i\;}-mean\left(x\right)\right)}^{2}}$, $p_{i}=\frac{x_{i}^{2}}{\sum_{i=1}^{N}x_{i}^{2}}$, $rms=sp_{2}$, $smr=sp_{8}$.

**Table 2:** General statistical parameters in frequency-space of AE signals.

$sp_{18}=\frac{X\times f^{T}}{\sum_{i=1}^{N}X_{i}}$	$sp_{19}=\sqrt{\frac{1}{N}\sum_{i=1}^{N}X_{i}{}^{2}}$
$sp_{20}=\sqrt{\frac{1}{N}\sum_{i=1}^{N}{\left(X_{i}-mean(X)\right)}^{2}}$	$sp_{21}=\sum_{i=1}^{N}X_{i}{}^{2}$

$X=\left[X_{1},\;X_{2},\ldots ,X_{N}\right]$ expresses a signal in frequency-space corresponding to the frequency vector **f**$=\left[f_{1},\;f_{2},\ldots ,f_{N}\right]$.

**Table 3:** Specifications of the wideband differential-auto sensor test (WDI-AST) sensors.

No	Parameters	Values
1	Peak sensitivity	96 dB
2	Operating frequency range	200–900 kHz
3	Directionality	+/−1.5 dB
4	Temperature range	−35 to 75 °C

**Table 4:** Details of the datasets employed to assess the offered method.

The Rate of Sampling = 1 MHz Signal Length = 1 s	Dataset 1		Dataset 2	
	Normal	0.3 mm	Normal	2.0 mm
Size of the training data	48	56	72	80
Size of the test data	12	14	18	20
Total	60	70	90	100

**Table 5:** Lists of signatures in order of best to worst together with their multivariable signature assessment coefficients (MSACs).

Grade	Dataset 1		Dataset 2	
	Signature	MSAC	Signature	MSAC
1	$sp_{8}$	0.4070	$sp_{18}$	0.1780
2	$sp_{18}$	0.1316	$sp_{8}$	−1.9828
3	$sp_{20}$	−0.2410	$sp_{11}$	−3.3668
4	$sp_{19}$	−0.4302	$sp_{19}$	−3.6743
5	$sp_{2}$	−0.4302	$sp_{2}$	−3.6743
6	$sp_{11}$	−0.7475	$sp_{21}$	−3.8037
7	$sp_{21}$	−0.8396	$sp_{13}$	−3.8037
8	$sp_{13}$	−0.8396	$sp_{20}$	−4.8188
9	$sp_{7}$	−3.4940	$sp_{15}$	−9.7089
10	$sp_{1}$	−5.1438	$sp_{16}$	−13.4133
11	$sp_{10}$	−5.4023	$sp_{7}$	−20.9734
12	$sp_{15}$	−5.8335	$sp_{6}$	−29.5249
13	$sp_{6}$	−6.5642	$sp_{1}$	−30.3707
14	$sp_{14}$	−8.6542	$sp_{10}$	−34.4620
15	$sp_{5}$	−8.6890	$sp_{9}$	−52.0857
16	$sp_{9}$	−8.7830	$sp_{3}$	−63.6919
17	$sp_{3}$	−8.8678	$sp_{14}$	−69.6629
18	$sp_{4}$	−8.8681	$sp_{5}$	−75.3590
19	$sp_{17}$	−9.4356	$sp_{4}$	−83.9089
20	$sp_{12}$	−12.4321	$sp_{17}$	−108.4910
21	$sp_{16}$	−12.5609	$sp_{12}$	−109.8144

**Table 6:** The classification accuracy results (%) for 10-fold cross validation.

No	Dataset 1			Dataset 2		
	All (%)	[12] (%)	The Proposed Method (%)	All (%)	[12] (%)	The Proposed Method (%)
1	100	92.31	100	89.47	84.21	89.47
2	100	76.92	84.62	78.95	84.21	89.47
3	100	100	100	84.21	84.21	84.21
4	92.31	92.31	92.31	100	100	100
5	100	92.31	92.31	78.95	84.21	84.21
6	100	69.23	92.31	100	100	100
7	100	100	100	94.74	94.74	100
8	100	92.31	92.31	100	100	100
9	100	100	100	94.74	94.74	94.74
10	100	92.31	100	100	100	100
Average	99.23	90.77	95.38	92.11	92.63	94.21

**Table 7:** Computational time comparison between the proposed and the conventional methods.

	Average Computational Time [Seconds]					
	All 21 Signatures Used for Leak Detection			The Proposed Method		
	Classifier Construction	Test	Total	Classifier Construction	Test	Total
Dataset 1	33.2924	8.1491	41.4415	22.9162	1.8927	24.8089
Dataset 2	48.2951	12.0008	60.2959	28.1876	2.7836	30.9712
